# An adaptive reflexive control strategy for walking assistance system based on functional electrical stimulation

**DOI:** 10.3389/fnins.2022.944291

**Published:** 2022-08-24

**Authors:** Hongtao Dong, Jie Hou, Zhaoxi Song, Rui Xu, Lin Meng, Dong Ming

**Affiliations:** ^1^Academy of Medical Engineering and Translational Medicine, Tianjin University, Tianjin, China; ^2^Department of Biomedical Engineering, College of Precision Instruments and Optoelectronics Engineering, Tianjin University, Tianjin, China

**Keywords:** functional electrical stimulation (FES), lower limbs, neurorehabilitation, gait assistance, adaptive reflexive control strategy

## Abstract

Functional electrical stimulation (FES) neuroprostheses have been regarded as an effective approach for gait rehabilitation and assisting patients with stroke or spinal cord injuries. A multiple-channel FES system was developed to improve the assistance and restoration of lower limbs. However, most neuroprostheses need to be manually adjusted and cannot adapt to individual needs. This study aimed to integrate the purely reflexive FES controller with an iterative learning algorithm while a multiple-channel FES walking assistance system based on an adaptive reflexive control strategy has been established. A real-time gait phase detection system was developed for accurate gait phase detection and stimulation feedback. The reflexive controller generated stimulation sequences induced by the gait events. These stimulation sequences were updated for the next gait cycle through the difference between the current and previous five gait cycles. Ten healthy young adults were enrolled to validate the multiple-channel FES system by comparing participants' gait performance to those with no FES controller and purely reflexive controller. The results showed that the proposed adaptive FES controller enabled the adaption to generate fitted stimulation sequences for each participant during various treadmill walking speeds. The maximum, minimum, and range of motion (ROM) of the hip, knee, and ankle joints were furtherly improved for most participants, especially for the hip and knee flexion and ankle dorsiflexion compared with the purely reflexive FES control strategy. The presented system has the potential to enhance motor relearning and promote neural plasticity.

## Introduction

Stroke is a neurological disorder with the world's highest prevalence. The number of patients with stroke in 2017 was over 100 million, which has almost doubled compared with 1990 (Arnao et al., 2016; Avan et al., 2019). About 80–90% of patients with stroke suffer from gait disorders, affecting their life quality and bringing heavy economic burdens to the patient's families and society (Schaechter, 2004; Hara, 2013; Shmuel et al., 2013; Mountain et al., 2020). Functional electrical stimulation (FES) is a technology that applies low-energy electrical pulses to the muscle resulting in active muscle contraction and further functional limb movements (Lynch and Popovic, 2008a). Electrical stimulation has been proven to increase muscle force, promote neuroplasticity, and enhance rehabilitation outcomes and is regarded as an effective rehabilitation treatment for gait disorders (Shin et al., 2022). However, most FES systems employ an open-loop control strategy with a constant stimulation mode in the market (Krishnamoorthy et al., 2008; Bulea et al., 2013; Chang et al., 2017). The open-loop control method has a simple computation and quick response advantage, but the constant stimulation mode cannot be adjusted for patients' assistance requirements in real-time. It may cause inadequate muscle activations and poor limb coordination.

The close-loop FES control strategy integrates feedback information, such as joint angles, electromyography (EMG), and human-machine interactive moment to adjust stimulation parameters based on the desired joint angle or moment trajectories. Seel et al. (2016) applied an iterative learning control (ILC) method to adjust the FES parameters based on inertial data to reduce muscle fatigue effectively. Jailani et al. (2010) proposed a knee biomechanical model that combined the joint trajectory control and fuzzy logic control, where the electrical pulse width was adjusted with the feedback of angle difference. However, as the human neuromuscular system is highly nonlinear (Dietz, 1992; Nielsen, 2002), the pure trajectory control model may not be readily applied to a real-time FES assistance system under various scenarios (Shiavi et al., 1987; Perry et al., 1995; Chen et al., 2018).

Some studies adopted biological-inspired control mechanisms in FES control strategies. Zhang et al. proposed a novel central pattern generator (CPG) based model to generate primary bipedal gaits in an FES walking system (Zhang et al., 2015). A long short-term memory (LSTM) neural network, proposed by Li et al. (2021), was used for predicting synchronous tibialis anterior (TA) EMG based on real-time angular velocity where the TA stimulation intensity was further modulated. Meng et al. proposed a purely reflexive control model to generate multiple electrical stimulation sequences (Meng et al., 2017). The gait events were mapped to muscle activity output during human walking. The model was realized to smooth limb coordination for walking assistance and reduce computational burden, making it straightforward to implement in practice. However, the stimulation parameters must be set before use and cannot be adjusted in real-time.

To further enhance the effectiveness of FES walking assistance, especially for meeting individuals' assistance needs under various walking speeds, we proposed a multiple-channel FES walking assistance system with an adaptive reflexive control method where the electrical stimulation parameters can be adjusted to temporal gait parameters and sagittal shank angle. A validation experiment was conducted by recruiting ten healthy young participants to walk on a treadmill at various speeds wearing the FES system. The gait performance under different stimulation control strategies (purely reflexive controller vs. adaptive reflexive controller) was compared and investigated.

## Methods

### Hardware design

**Figure 1:**
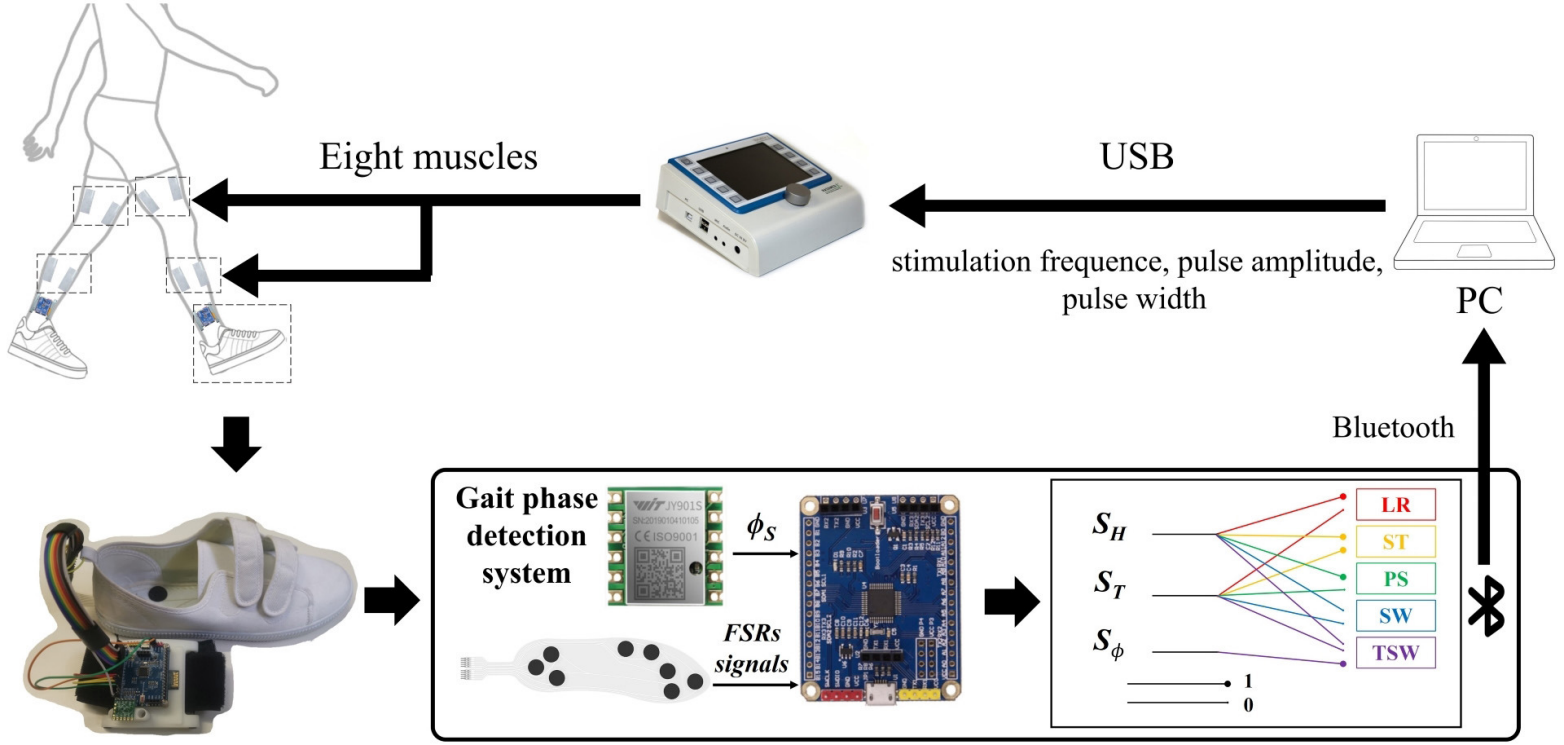
The structure of the functional electrical stimulation (FES) walking assistance system. The system consists of three main parts: a programmable electrical stimulator, a real-time gait phase detection device, and a host computer. The FSR-embedded insole and an inertial measurement unit (IMU) chip were connected to an STM32 microcontroller that detected gait phases by an IF-THEN type finite state machine during walking. The sensory signals, including gait phase detection result and shank angle, were transmitted to the host PC *via* Bluetooth. At the same time, the adaptive FES controller generated electrical stimulation sequences for the muscles. The stimulation parameters were adapted and applied to eight muscles by the programmable electrical stimulator RehaStim through a USB 3.1 port.

As shown in Figure 1, the FES system consists of a self-designed real-time gait phase detection system, an 8-channel programmable electrical stimulation device (RehaStim 2, HASOMED GmbH, Germany), and a host computer (Intel 6 Core i7-8750H, 2.20 GHz, and Windows 10 system).

The wearable real-time gait phase detection system includes force-sensitive resistors (FSR) embedded in shoe insoles and an inertial measurement unit (IMU; JY901, Witmotion, Shenzhen, China), as shown in Figure 1. The 6-axis IMU consists of an accelerometer and a gyroscope measuring acceleration and angular rate along three orthogonal axes. The STM32 chip (STM32F103C8T6, Witmotion, Shenzhen, China) is used for data acquisition, gait event detection, and communication with the host computer *via* Bluetooth 2.0.

The algorithms described in the following sections have been implemented in a C++ program and Qt software. The RehaStim 2 consists of eight electrical stimulation channels based on two separately controlled modules and is connected to the host computer through a USB 3.1. The electrical stimulation parameters, such as stimulation frequency, pulse width (PW), and pulse amplitude, can be controlled by the host computer *via* the ScienceMode2 communication protocol in real-time.

### FES reflexive control strategy

**Figure 2:**
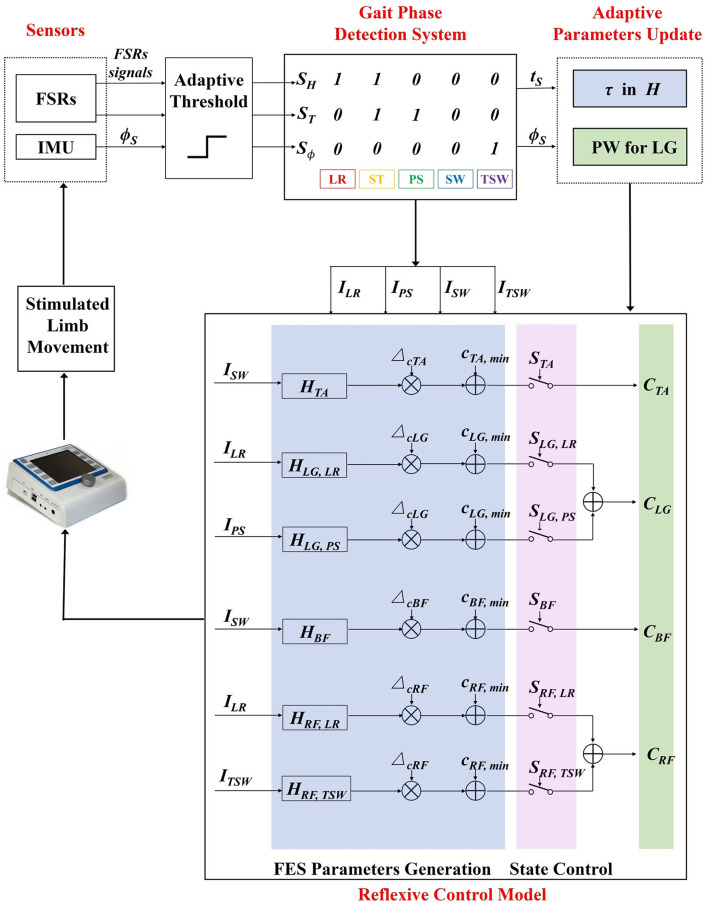
The adaptive reflexive FES control diagram. Input signals are first translated into binary signals by an adaptive threshold. Gait phases are identified based on a rule-based machine learning algorithm. Gait event impulses are generated when specific gait transitions between gait phases occur. A hierarchical FES control model consists of two levels of control where the top level switches the stimulation state of muscles, and the low level generates the stimulation sequences. The time constant parameter τ and stimulation pulse width are adaptively updated with an iterative learning method in the low level controller based on the real-time feedback of muscle stimulation time *t*_*s*_ and sagittal shank angle ϕ_*TO*_, respectively. LR, load response; ST, stance; PS, pre-swing; SW, swing; TSW, terminal swing.

Four muscles were selected for each leg, namely, tibialis anterior (TA), lateral gastrocnemius (LG), biceps femoris (BF), and rectus femoris (RF). The muscles are associated with the flexion/extension of the hip, knee, and ankle during walking. The reflexive control strategy generates stimulation sequences of eight muscles based on the event impulses from the gait phase detection system. A hierarchical FES controller is shown in Figure 2. The top level employs a finite state control model where the state function *S* switches on and off the stimulation of muscles for movement coordination. In the low level, the transfer function *H* generates the impulse responses for stimulation amplitude by convolving with an event impulse.

The transfer function *H* is a second-order low-pass Bessel filter, as shown below.


$$\begin{array}{l} H\left(t\right)=\left(g\frac{1}{\tau }\;e^{\frac{-1.5t}{\tau }}\sin\left(\frac{\sqrt{3}t}{2\tau }\right)\right) \end{array}$$

The τ is the time coefficient derived from the filter cut-off frequency and determines the profile of the impulse response. The *g* is the gain coefficient, normalizing the impulse response to 0 and 1.

The state functions are responsible for switching on/off the electrical stimulation according to five detected gait phases: load response (LR), stance (ST), pre-swing (PS), swing (SW), and terminal swing (TSW).


$$\begin{array}{l} S_{TA}\;=\{\begin{array}{cc} 1, & state=SW\;or\;TSW\\ 0, & otherwise \end{array}\\ S_{BF}\;=\{\begin{array}{cc} 1, & state=SW\;or\;TSW\\ 0, & otherwise \end{array}\\ S_{LG,LR}\;=\{\begin{array}{cc} 1, & state=LR\\ 0, & otherwise \end{array}\\ S_{LG,PS}\;=\{\begin{array}{cc} 1, & state=PS\\ 0, & otherwise \end{array}\\ S_{RF,LR}\;=\{\begin{array}{cc} 1, & state=LR\\ 0, & otherwise \end{array}\\ S_{RF,TSW}=\{\begin{array}{cc} 1, & state=TSW\\ 0, & otherwise \end{array} \end{array}$$

**Figure 3:**
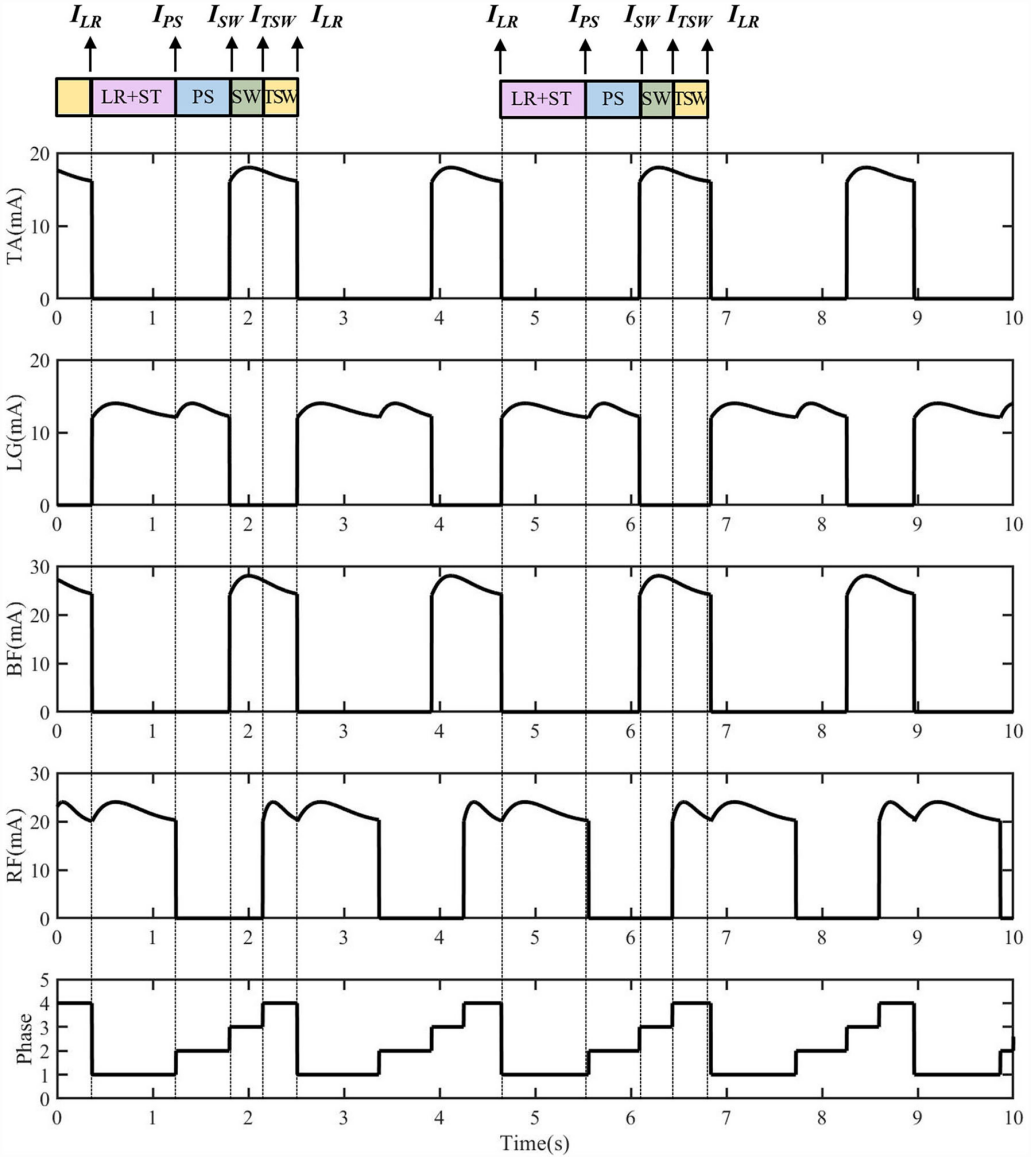
The electrical stimulation sequences for four muscles of one side generated from the reflexive FES controller. The **I**_**LR**_ triggered the lateral gastrocnemius (LG) and rectus femoris (RF) muscle activations for the knee extension and hip flexion. The **I**_**PS**_ triggered the LG muscle for ankle plantarflexion. The **I**_**SW**_ triggered the tibialis anterior (TA) and biceps femoris (BF) muscles for the ankle dorsiflexion, knee flexion, and hip extension at early swing. The **I**_**TSW**_ triggers TA, RF, and BF muscles for the preparation of load response.

The FES sequences of eight muscles generated by the reflexive controller during treadmill walking are shown in Figure 3. The generation of electrical stimulation patterns elicited by impulse signals for each muscle is expressed as follows:


$$\begin{array}{l} C_{TA}=\left(H_{TA,LR}\;*\;I_{SW}\cdot \Delta_{cTA}\;+\;C_{TA,\min}\right)\cdot S_{TA}\\ C_{LG}=\left(H_{LG,LR}\;*\;I_{LR}\cdot \Delta_{cLG}\;+\;C_{LG,\min}\right)\cdot S_{LG,LR}\\ \;+\;\left(H_{LG,PS}\;*\;I_{PS}\cdot \Delta_{cLG}\;+\;C_{LG,\min}\right)\cdot S_{LG,PS}\\ C_{BF}=\left(H_{BF,SW}\;*\;I_{SW}\cdot \Delta_{cBF}\;+\;C_{BF,\min}\right)\cdot S_{BF}\\ C_{RF}=\left(H_{RF,LR}\;*\;I_{LR}\cdot \Delta_{cRF}\;+\;C_{RF,\min}\right)\cdot S_{RF,LR}\\ \;+\;\left(H_{RF,TSW}\;*\;I_{TSW}\cdot \Delta_{cRF}\;+\;C_{RF,\min}\right)\cdot S_{RF,TSW} \end{array}$$

where, *I* is the gait event impulse generated from gait phase transitions, *and H* is the transfer function that generates the response output by convolving with the impulse input *I*. The Δ_*c*_ is the difference between *C*_max_ and *C*_min_ where *C*_max_ is the maximum threshold current amplitude that can produce a maximal muscle contraction without any discomfort, and *C*_min_ is the minimum threshold current amplitude that can elicit a visible muscle contraction. The values of *C*_max_ and *C*_min_ for each muscle were measured in a preparation experiment for every participant, detailed in Appendix 1 document.

### Gait phase detection

An IF-THEN type finite state machine was employed to detect five gait events, namely, heel strike (HS), foot flat (FF), heel off (HO), toe off (TO), and sagittal threshold angle (STA). These gait events are furtherly used to define gait phases, such as LR, ST, PS, SW, and TSW. The sensory signals include foot contact signals from FSRs and angle signals from the IMU attached to the shank. An adaptive threshold method is used to convert the inputs to binary signals. The *S*_*H*_ and *S*_*T*_ are binary signals representing the heel and toe contact states where the logic value of 1 indicates that the heel or toe is in contact with the ground, and 0 indicates that it is off the ground. The binary signal *S*_ϕ_ represents the state of sagittal shank angle (ϕ_*S*_) during the swing phase (*S*_*H*_ = *S*_*T*_= 0). It determines the initiation of TSW when a participant extends the knee to prepare to strike the foot on the floor. Four types of gait impulses, *I*_*LR*_, *I*_*PS*_, *I*_*SW*_, and *I*_*TSW*_ are generated for the FES controller based on gait phase transitions, as shown in Figure 2.

*I*_*LR*_: the impulse indicates the initial foot contact with the ground. In normal gait, the heel usually strikes the ground first. However, individuals with a pathological walk may establish foot contact with the forefoot. Therefore, the transition is detected if any foot part touches the ground after the swing phase (last state: *S*_*H*_ = 0, *S*_*T*_ = 0; and current state: *S*_*H*_ = 1 or *S*_*T*_ = 1).

*I*_*PS*_:the transition occurs when the FSR underneath the heel is not pressed, and the forefoot is still in contact with the ground. This event indicates a transition from the stance phase to the pre-swing phase (last state: *S*_*H*_ = *S*_*T*_ = 1; and current state: *S*_*H*_ = 0, *S*_*T*_ = 1).

*I*_*SW*_:the impulse indicates the transition from the stance or pre-swing phase to the swing phase, where the swing phase is when the foot is lifted entirely off the ground so that no FSRs are pressed (last state: *S*_*H*_ = 1 or *S*_*T*_ = 1; and current state: *S*_*H*_ = *S*_*T*_ = 0).

*I*_*TSW*_:the impulse indicates the transition from the swing phase to the terminal swing phase when the hip flexes forward and the measured ϕ_*S*_ reaches its threshold (last state: *S*_*H*_ = *S*_*T*_ = 0, *S*_ϕ_ = 0; and current state: *S*_*H*_ = *S*_*T*_= 0, *S*_ϕ_ = 1).

### Adaptive parameters update

An adaptive method is proposed where the muscle stimulation time *t*_*s*_ and sagittal shank angle at TO (ϕ_*TO*_) are used as real-time feedback signals to meet the assistance needs of various gait speeds. The adaptive model updates the time coefficient parameter τ of eight muscles and PW of both LG muscles. The time constant determines the stimulation amplitude profile while the PW of LG muscles modulates the ankle push-off at various speeds (Brockett and Chapman, 2016). These parameters are updated based on the muscle stimulation time and sagittal shank angle of the previous five gait cycles. One gait cycle is regarded as the interval between consecutive heel strikes of the same foot (*S*_*H*_ =1, *S*_*T*_ = 0).

According to the difference in the stimulation duration time between the previous five gait cycles and the current gait cycle, the closed-loop control model adjusts the corresponding τ to change the muscle stimulation time for the next gait cycle. Take τ_*TA*_ as an example.

The stimulation of TA is activated during the SW and TSW, as shown in Figure 3. If the stimulation time of TA in the current gait cycle is *t*_*TA*_(*n*), the average stimulation time of the previous five gait cycles can be calculated as ${\bar{t}}_{TA}$:


$$\begin{array}{l} {\bar{t}}_{TA}=\frac{1}{5}\overset{5}{\underset{i=1}{\sum }}t_{TA}\left(n-i\right) \end{array}$$

The difference between the *t*_*TA*_ and ${\bar{t}}_{TA}$ can be calculated as $\Delta t_{TA}=t_{TA}\left(n\right)-{\bar{t}}_{TA}$. If |Δ*t*_*TA*_| > 0.04 s, the time coefficient τ_*TA*_ is updated as follows:


$$\tau_{TA}\left(n+1\right)=\{\begin{array}{c} \tau_{TA}\left(n\right)+L\;\Delta t_{TA}<-0.04s\\ \tau_{TA}\left(n\right)-L\;\Delta t_{TA}>0.04s \end{array}$$

where, τ_*TA*_(*n*+1) is the transfer function time coefficient of the next gait cycle and τ_*TA*_(*n*) is the transfer function time coefficient of the current gait cycle. As the response time of the transfer function fitted by a second-order low-pass Bessel filter is about one-fourth of the overall activation time, the update threshold is set as 0.04 with an iterative learning step *L* of 0.01. In addition, the value of τ is limited between 0.01 and 1 due to the requirement of muscle response time for movement coordination during human walking.

According to the second-order low-pass Bessel filter properties of the transfer function *H*_*TA, TO*_, the cut-off frequency *f*_*c*_ can be calculated based on the time coefficient:


$$\begin{array}{l} f_{c}=\frac{1}{2\pi \;*\;\tau } \end{array}$$

**Figure 4:**
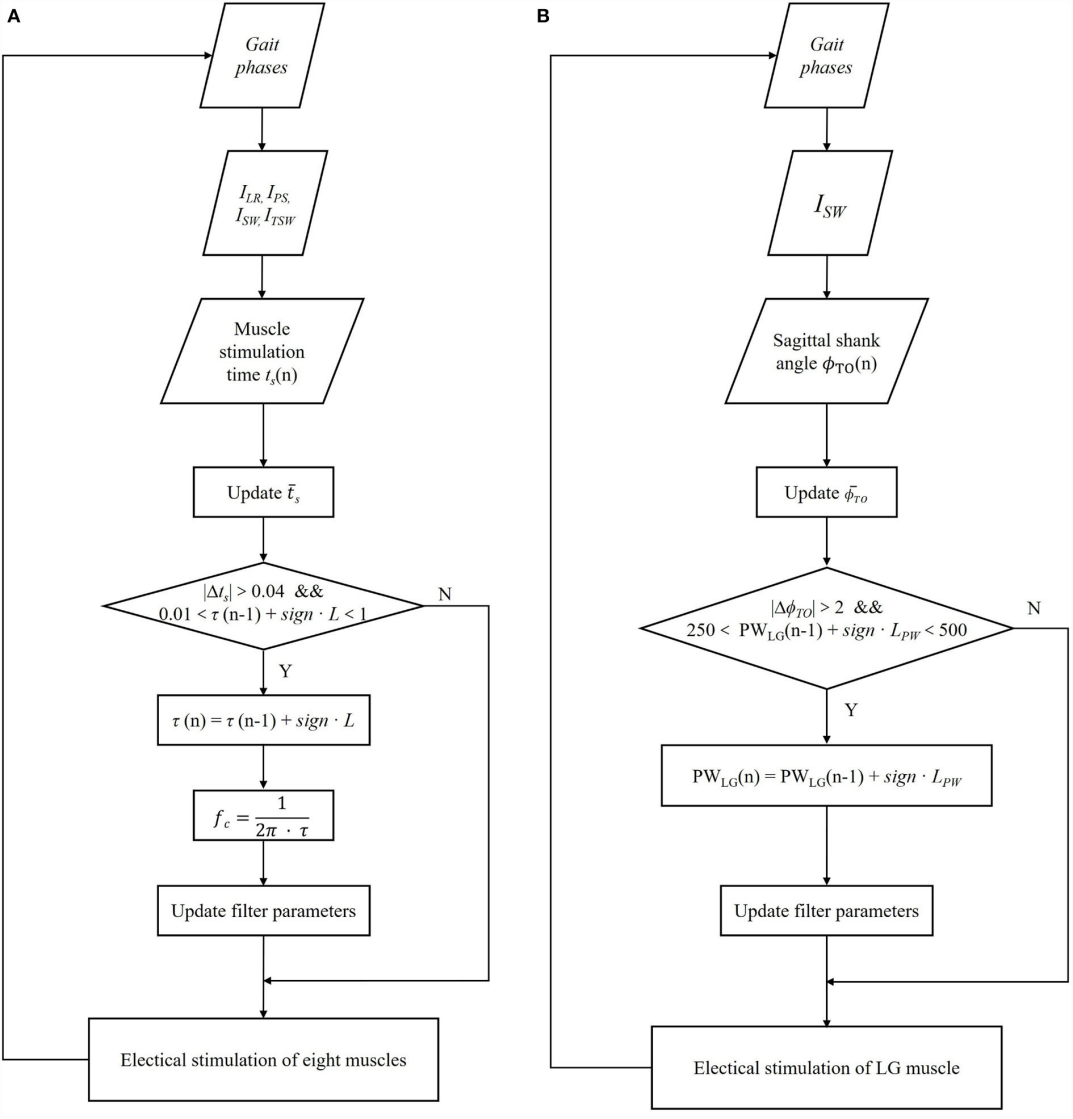
Block diagram of the iterative learning control algorithm for transfer function time constant coefficient τ of all eight muscles **(A)** and pulse width parameter of two lateral gastrocnemius muscles (LG) **(B)** based on each gait cycle.

Eventually, the cut-off frequency *f*_*c, TA, TO*_ is updated to the adaptive reflexive controller for adjusting the amplitude stimulation profile of *C*_*TA*_. The same procedure is applied to all muscles. The update progress is shown in Figure 4A.

Similarly, according to the ${\bar{\phi }}_{TO}$ of previous five gait cycles and the ϕ_*TO*_ of the current gait cycle, the PW values of both LG of the next gait are interactively updated:


$$PW_{LG}\left(n+1\right)=\{\begin{array}{c} PW_{LG}\left(n\right)+L_{PW}\;\Delta \phi_{TO}<-2^{{}^{\circ}}\\ PW_{LG}\left(n\right)-L_{PW}\;\Delta \phi_{TO}>2^{{}^{\circ}} \end{array}$$

where, *PW*_*LG*_(*n*+1) is the PW of LG muscle for the next gait cycle, *PW*_*LG*_(*n*) is the stimulation pulse width at the current gait. $\Delta \phi_{TO}=\phi_{TO}\left(n\right)\;-\;{{\bar{\phi }}_{TO}}_{\;}$. The step *L*_*PW*_ of iterative learning is set to 20 μs. Additionally, the limited range of *PW*_*LG*_ is set between 250 and 500 μs. The update process is shown in Figure 4B.

## Experiment

### Experimental set-up

For this experiment, ten healthy young adults (ten men) were recruited. The mean [±standard deviation (SD)] age was 25.1 (±1.6) years, and the mean (±SD) height was 177.3 (±5.83) cm, as shown in Table 1. The participants were fully informed of the procedure and gave written consent before the experiment. The study was approved by the Ethics Committee of Tianjin University and was conducted in the Motion Rehabilitation Laboratory of Tianjin University.

**Table 1:** Participants' demographic information.

**Subjects**	**Gender**	**Age (years)**	**Height (cm)**	**Weight (kg)**
A	M	26	174	80
B	M	27	175	78
C	M	26	176	76
D	M	25	181	67
E	M	25	170	69
F	M	25	181	60
G	M	24	188	78
H	M	26	185	85
I	M	26	172	70
J	M	21	171	62

M, male.

**Figure 5:**
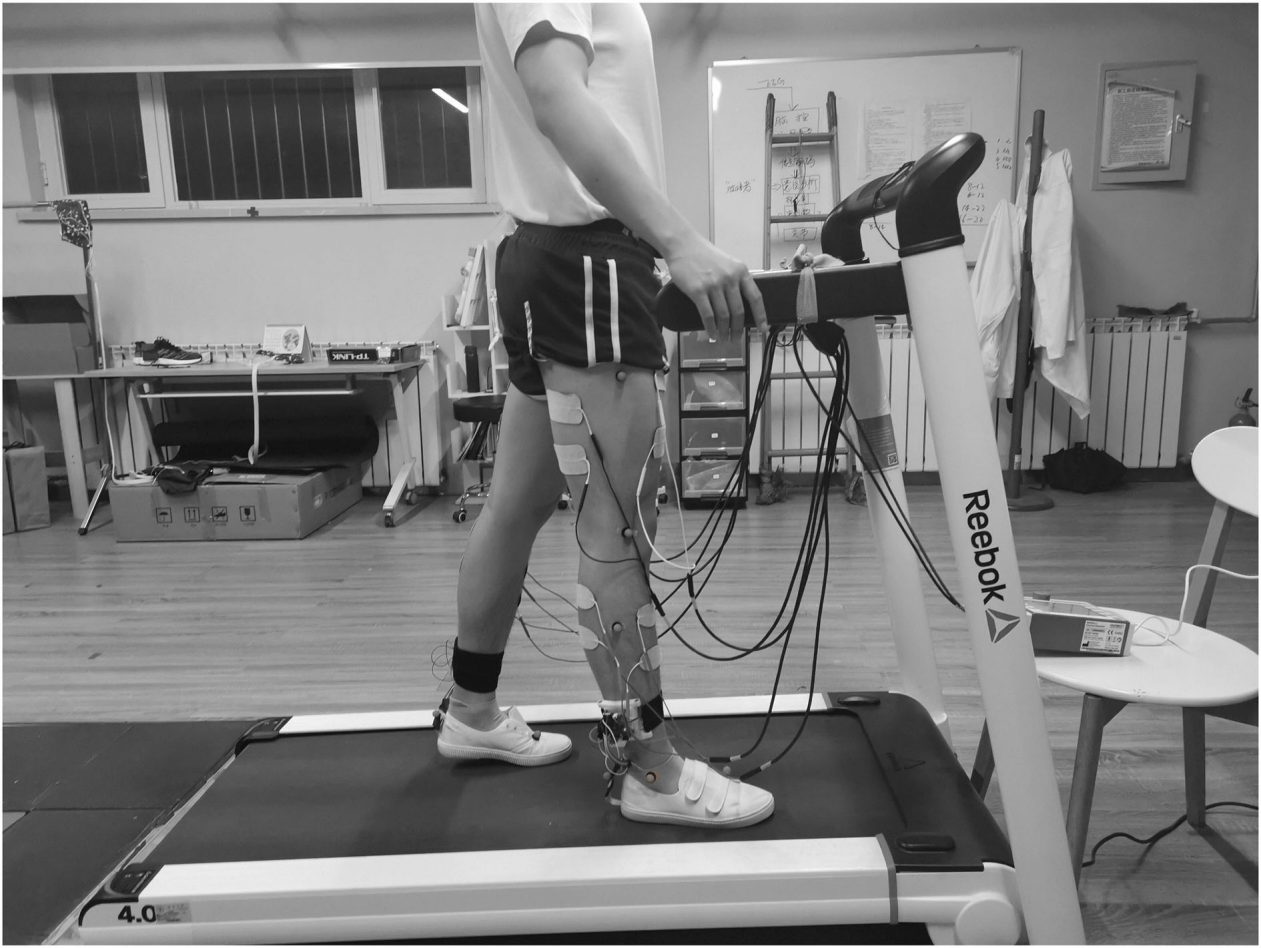
Schematic of the experimental setup: a participant walked on the treadmill wearing real-time gait detection devices. Electrical stimulation electrodes were attached to the eight muscles, and the retroreflective markers were placed on the lower limbs.

Eight muscles were selected in the experiment: RF, BF, LG, and TA of both legs, to augment hip, knee, and ankle flexion/extension, respectively. Electrical stimulation electrodes were placed on the muscles, and the *C*_max_ and *C*_min_ of every muscle were measured in the preparation session. The measurement procedure and results are shown in Appendix 1 document. Participants wore shorts and gait detection devices. The Vicon Plug-in-Gait (PiG) model was used to evaluate the gait performance of the participants where retroreflective markers were attached to the anterior superior iliac spine, the posterior superior iliac spine, thigh, knee, ankle, tibial wand, heel, and toe of both sides, as shown in Figure 5.

The participants were instructed to walk on a treadmill under three different conditions: (1) without FES controller (NFC); (2) with a purely reflexive FES controller (RFC); and (3) with an adaptive reflexive FES controller (ARFC). In each session, the treadmill walking speed increased from 1.0 to 2.0 km/h and then decreased to 1.0 km/h with an incrementation step of 0.2 km/h. The participants needed to complete at least 15 gait cycles at each speed. The host computer collected stimulation parameters, detected gait phases, and shank angle information while the Vicon Nexus software captured marker trajectories with a sampling rate of 100 Hz. The Vicon Lock Sync device was used to synchronize the collected data.

### Data analysis

Joint kinematic data of the hip, knee, and ankle were obtained using the PiG model. One gait cycle data were time-normalized to 0–100% with 101 samples. A total of 165 gait cycles were extracted for each participant. The maximum and minimum of the hip, knee, and ankle were calculated based on gait cycles and investigated using a one-way analysis of variance (ANOVA) with stimulation pattern as the main factor. A paired t-test was performed to evaluate the difference in gait kinematics under three stimulation conditions. All statistical analyses were performed using the MATLAB Statics Toolbox (MATLAB2020a, The MathWorks, USA). Statistical significance was set as *p* < 0.05.

## Results

**Figure 6:**
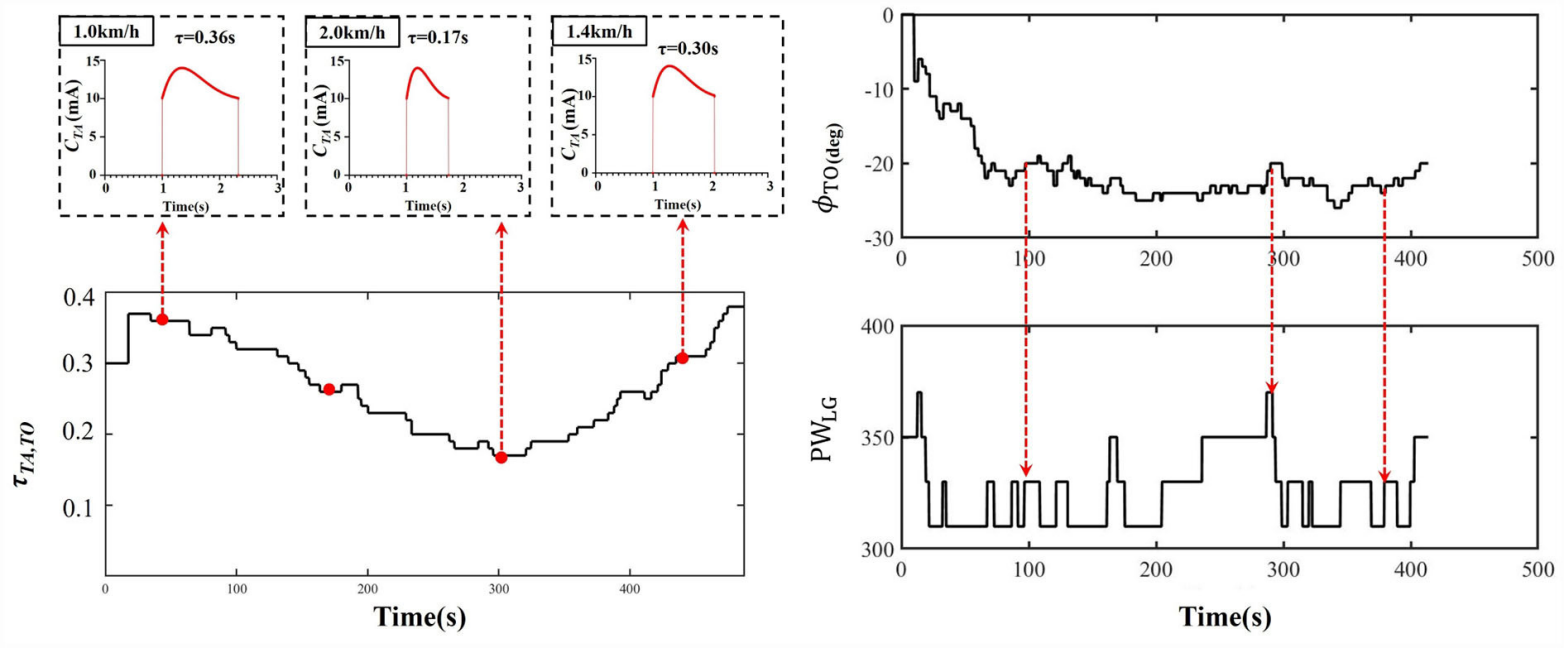
An example of the time constant coefficient τ_*TA, TO*_ of TA muscle and pulse width (PW) of LG muscle over in a trial of one participant with walking speed varying from 1.0 to 2.0 km/h.

Table 2 showed that the real-time gait event detection algorithm obtained an accuracy rate of 100% to identify all five gait phases, and the average delay time was less than 20 ms. The FES sequences for eight muscles were generated from the transfer functions triggered by the gait event impulses during treadmill walking, as shown in Figure 3. Figure 6 shows that the time coefficient τ_*TA, TO*_ and pulse width of LG muscles were adaptively adjusted with various walking speeds during one participant's trial. We can see that the rise time of the stimulation pattern responded more quickly at 2.0 km/h speed compared with those at slower speeds indicating that the ARFC can efficiently adjust the stimulation pattern according to the change in walking speeds.

**Table 2:** Accuracy and time latency for gait event detection during various speeds.

**Speed (km/h)**	**Total stride number**	**Gait events**	**Accuracy (%)**	**Average latency (ms)**
1.0 ~ 1.4	900	HS	100	9.6
		HO	100	14.4
		TO	100	13.9
		STA	100	10.5
1.6 ~ 2.0	750	HS	100	10.1
		HO	100	21.4
		TO	100	15.0
		STA	100	17.3

HS, heel strike; HO, heel off; TO, toe off; STA, the shank angle _ϕ_S__reached its threshold during the swing.

**Figure 7:**
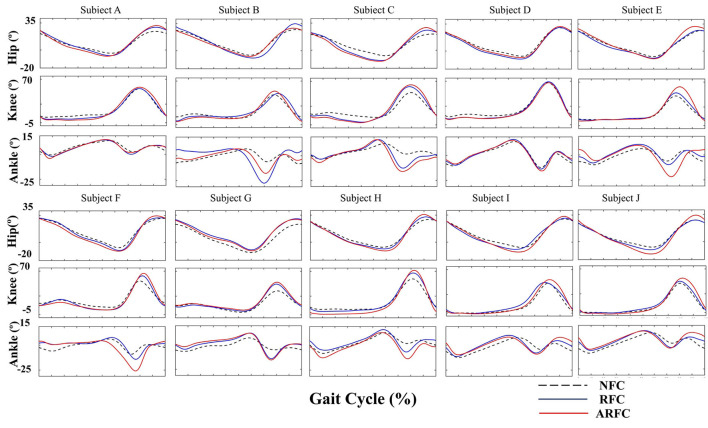
Joint kinematics of the hip, knee, and ankle joints under three different stimulation conditions for individual participants.

Joint kinematics were compared under three stimulation conditions for all participants, as shown in Figure 7. Results showed that both stimulation control strategies (RFC and ARFC) did not hinder normal gait patterns (Figure 7) and significantly promoted the joint movement, as shown in Figure 7 and Table 3. The participants achieved larger flexion and extension of the hip and knee as the electrical stimulation applied to the RF and BF muscles helped in promoting the joint movement (Table 3). The electrical stimulation on the TA muscle led to a higher ankle maximum angle than that without FES assistance. It can also be observed that the ARFC has a better promoting effect than the RFC in all joint kinematic parameters. The participants obtained a larger ROM of the hip (RFC: 35.16 ± 3.92; ARFC: 37.85 ± 4.99), knee (RFC: 54.34 ± 8.05; ARFC: 59.54 ± 8.20), and ankle (RFC: 22.44 ± 6.52; ARFC: 25.07 ± 6.36) with the ARFC compared with the RFC. The ankle push-off at the terminal stance was also increased during the ARFC trial (RFC: −10.81 ± 7.76; ARFC: −13.92 ± 7.95). The results indicated that the proposed ARFC method could provide better gait assistance at different speeds.

**Table 3:** Effects of stimulation condition on joint kinematic parameters using one-way analysis of variance (ANOVA).

**Kinematic parameters (°)**	**NFC**	**RFC**	**ARFC**	**F-value**	***P*-value**
Hip maximum	23.69 ± 3.40	26.26 ± 3.24	27.87 ± 3.17	679.4	<0.0001
Hip minimum	−6.60 ± 2.78	−8.87 ± 2.501	−10.04 ± 3.19	620.5	<0.0001
Hip ROM	30.28 ± 4.07	35.16 ± 3.92	37.85 ± 4.99	1264	<0.0001
Knee maximum	53.23 ± 9.27	58.44 ± 8.59	62.62 ± 7.55	502.8	<0.0001
Knee minimum	6.59 ± 5.11	4.14 ± 3.82	3.16 ± 4.30	258.4	<0.0001
Knee ROM	46.64 ± 8.46	54.34 ± 8.05	59.54 ± 8.20	1016	<0.0001
Ankle maximum	8.47 ± 2.73	10.74 ± 2.78	10.40 ± 3.38	275.1	<0.0001
Ankle minimum	−7.45 ± 3.82	−11.69 ± 6.89	−14.55 ± 7.15	549.7	<0.0001
Ankle ROM	15.93 ± 4.00	22.44 ± 6.52	25.07 ± 6.36	1088	<0.0001
Ankle minimum during TS	−2.35 ± 5.22	−10.81 ± 7.76	−13.92 ± 7.95	1165	<0.0001

NFC, no FES controller; RFC, reflexive FES controller; ARFC, adaptive reflexive FES controller.

## Discussion

The FES is an effective technique to restore gait functions for patients with motor disorders (Lynch and Popovic, 2008b; Popovic, 2014). Due to the disturbances from internal time-varying muscle characteristics with electrical stimulation and external environmental uncertainties, most current FES systems used pre-set stimulation patterns and parameters and mainly focused on the drop foot correction. Patients may not achieve satisfactory gait performances due to the low adaptability of FES control strategies (Krishnamoorthy et al., 2008; Bulea et al., 2013; Chang et al., 2017). Therefore, accurate gait phase detection and adaptive control strategy are the critical parts of high adaptability to provide efficient walking assistance and rehabilitation.

A wearable real-time gait phase detection device integrating the FSRs-embedded shoe insole and IMU was developed. The reliability and feasibility of the combination of the FSRs and IMU in gait phase detection have been proved in previous studies (Prasanth et al., 2021). The FSRs can provide the most reliable information about foot contact conditions (Hanlon and Anderson, 2009), and the data from the inertial sensor added the information during the swing. Therefore, the combination of FSRs and IMU enables the identification of multiple gait phases during a gait cycle. Pappas et al. (2001) reported above 96% detection accuracy of HS, FF, HO, and TO for both unimpaired and pathological gait with a detection delay of less than 90 ms using a threshold-based method. Sui et al. (2020) proposed a Convolutional Neural Network (CNN)-based gait detection algorithm, which achieved an average error of 8.86 ms for the TO detection and 9.12 ms for the HS, and a gait phase detection accuracy of 96.44% on healthy subjects. Our study proposed a rule-based machine learning algorithm for identifying five gait phases: HS, FF, HO, TO, and STA. The real-time performance achieved an accuracy rate of 100% and an average delay of less than 20 ms.

Multiple-channel FES systems with adaptive control methods were proposed in previous studies (Ladouceur and Barbeau, 2000; Johnston et al., 2003; Kesar et al., 2011; Street et al., 2015; Miller et al., 2016; da Cunha et al., 2021). Mueller et al. (2020) proposed an FES system-based ILC in which individual fitted stimulation patterns of the antagonistic muscle pairs for the knee and ankle joints were generated by warping healthy subjects' physiological joint angles trajectories. The experimental results showed slight improvements in the peak joint angles in the range of 4 degrees on three of four spinal cord injured subjects. Jiang et al. (2020) proposed an adaptive FES control method that employed a linear model with ILC to adjust the stimulation timing and intensity according to the average walking speed and the error between the actual maximum ankle dorsiflexion and target angle. Their proposed control method obtained a better orthotic effect for foot drop correction than the performance with constant pre-set stimulation parameters. However, these FES control strategies required complex calibration procedures and complicated mathematical models. Compared with these complicated models, the biological-inspired FES strategies have shown their advantages in motor relearning and simplicity in modeling (Meng et al., 2017). However, the purely RFC cannot adjust the stimulation pattern for participants' individual needs during various walking speeds. This study was the first attempt to integrate the purely RFC and iterative learning algorithm and develop a multiple-channel FES walking assistance system based on an adaptive reflexive control strategy. An adaptive algorithm based on the iterative learning method was proposed to adjust the electrical stimulation parameters corresponding to muscle stimulation time and sagittal shank angle. A multiple-channel FES system was established, and the validation experiment was performed by recruiting healthy young adults. The results showed that the ARFC method achieved a better promoting effect on joint kinematics during treadmill walking than the pure RFC controller at various speed conditions.

The functionality of the ARFC was evaluated in a validation experiment involving ten healthy young male participants compared with their gait performance under the NFC and RFC stimulation conditions. The participants did not report any discomfort or disturbance during treadmill walking with the stimulation applied. The FES control strategy provided a correct muscle activation sequence consistent with the participant's voluntary movements. The ARFC significantly improved the maximum, minimum, and ROM for most participants compared with the RFC. The ankle plantarflexion angle using the ARFC was significantly larger than the RFC, indicating that the adaptive change of time constant coefficient τ and PW increased the ankle push-off and further promoted walking speed. The ankle plantarflexion and knee flexion play a critical role in generating forward propulsion (Neptune et al., 2001; Anderson et al., 2004), and the patients often exhibited a reduction in the ankle and knee movements (Bhadra et al., 2001; Kesar et al., 2010). The ARFC also achieved a larger knee and hip flexion angle in early swing, which would help improve foot clearance and leg swing. It may provide more appropriate training assistance for patients with a neurological disease with individual stimulation pattern adjustment and potentially enhance motor learning and promote neural plasticity.

There are still some limitations in this study. The experiment only recruited healthy young men, and the subject size was relatively small. The enrolled healthy young subjects have intact motor units and good muscle responses to electrical stimulation compared to patients with stroke who usually have muscular atrophy combined with a damaged perception level (Arasaki et al., 2009; Shin et al., 2022). The affected muscular properties might have a potential influence on the modulation of the stimulation parameters and hinder the performance of the FES assistance (Ambrosini et al., 2014). Moreover, we did not observe effective PW parameter adjustment for LG muscles during treadmill walking. It might be because healthy participants can meet the propulsion need of ankle plantarflexion by voluntary LG muscle contraction, and the scenario of treadmill walking limits the participants' walking variation. The proposed multiple-channel FES needs further validation with stroke patients, and the overground walking experiment should be considered in future studies.

## Conclusion

This article proposed a multiple-channel FES walking assistance system with an adaptive reflexive FES control strategy. The validation experiment was performed by recruiting ten healthy young men. Walking performance under three stimulation conditions was investigated and compared. The results showed that the system generates accurate stimulation patterns for each muscle group while the stimulation parameters were successfully further adapted to various walking speeds. The ARFC method significantly improved the maximum, minimum, and ROM of the hip, knee, and ankle joints, especially for the hip and knee flexion and ankle dorsiflexion, compared with the purely RFC strategy. The presented system has the potential to provide efficient gait assistance for patients and promote motor relearning and neural plasticity. Future studies will carry out a clinical experiment to prove the system effect's on patients with stroke.

## Data availability statement

The datasets presented in this article are not readily available as the study is still ongoing. Requests to access the datasets should be directed to linmeng@tju.edu.cn.

## Ethics statement

The studies involving human participants were reviewed and approved by the Ethics Committee of Tianjin University. The patients/participants provided their written informed consent to participate in this study. Written informed consent was obtained from the individual(s) for the publication of any potentially identifiable images or data included in this article.

## Author contributions

LM and DM: conception and design of the study. HD, JH, and ZS: system development and experiments. HD and JH: analysis and interpretation of data and drafting the manuscript. LM: project supervision and manuscript revision. RX: revising the article critically for important intellectual content. DM: project administration. All authors approved the final version to be submitted.

## Funding

The work was funded by the National Key R&D Program of China (2020YFC2004300 and 2020YFC2004302), the National Natural Science Foundation of China (82001921), and the Natural Science Foundation of Tianjin (20JCZDC0080).

## Conflict of interest

The authors declare that the research was conducted in the absence of any commercial or financial relationships that could be construed as a potential conflict of interest.

## Publisher's note

All claims expressed in this article are solely those of the authors and do not necessarily represent those of their affiliated organizations, or those of the publisher, the editors and the reviewers. Any product that may be evaluated in this article, or claim that may be made by its manufacturer, is not guaranteed or endorsed by the publisher.
