# Supplementary material for: An adaptive reflexive control strategy for walking assistance system based on functional electrical stimulation
Source: Front Neurosci. 2022 Aug 24;16:944291. doi: 10.3389/fnins.2022.944291 (PMC9450861; doi:10.3389/fnins.2022.944291)
Supplement: Supplementary file 1 [file Table_1.DOCX]

Appendix

# FES setup

Eight muscles were selected in the study: RF, BF, LG, and TA for both legs to augment hip, knee, and ankle flexion/extension movements. Prior to the experiment, the $C_{max}$ and $C_{min}$ of each muscle were measured. The procedure is as follows:

1. All electrodes were carefully placed at the appropriate anatomical locations to produce sufficient muscle contraction of the desired muscles.
2. The initial PW was set to 350us and the stimulation frequency was 40Hz. The current amplitude was increased from 0 mA by a increment step of 1mA while the muscle contraction was observed and the subjects were asked whether they had pain or discomfort.
3. When the stimulated muscle generated visible contraction and the patient had no discomfort or pain, the current value of the electrical stimulation was recorded as $C_{min}$.
4. When the stimulated muscle generated maximum muscle contraction and the patient had no discomfort or pain, the current value of the electrical stimulation was recorded as $C_{max}$. It needs to be noted that the actual maximal value used in FES assisted trial was the $C_{max}$multiplied by 0.9 coefficient.

The FES parameters were detailed in Table 1 for each subject as shown below.

Table 1 Stimulation parameters determined in the FES setup. Eight muscles were chosen in the study, namely, the TA, LG, BF, and RF for each leg. The parameters $\boldsymbol{C}_{\boldsymbol{max}}$ and $\boldsymbol{C}_{\boldsymbol{min}}$ were measured for each muscle. The units are mA.

| **Subjects** | **Current(mA)** | **LTA** | **LLG** | **LBF** | **LRF** | **RTA** | **RLG** | **RBF** | **RRF** |
| --- | --- | --- | --- | --- | --- | --- | --- | --- | --- |
| **A** | ***C_min_*** | 8 | 10 | 12 | 14 | 8 | 8 | 14 | 16 |
|  | ***C_max_*** | 12 | 12 | 18 | 20 | 12 | 12 | 22 | 20 |
| **B** | ***C_min_*** | 10 | 8 | 18 | 16 | 8 | 8 | 18 | 16 |
|  | ***C_max_*** | 12 | 10 | 24 | 18 | 14 | 10 | 24 | 20 |
| **C** | ***C_min_*** | 10 | 12 | 16 | 18 | 10 | 12 | 16 | 20 |
|  | ***C_max_*** | 14 | 16 | 22 | 22 | 14 | 16 | 20 | 22 |
| **D** | ***C_min_*** | 14 | 18 | 14 | 14 | 14 | 18 | 14 | 14 |
|  | ***C_max_*** | 16 | 22 | 16 | 20 | 18 | 20 | 16 | 20 |
| **E** | ***C_min_*** | 10 | 10 | 16 | 14 | 10 | 10 | 16 | 12 |
|  | ***C_max_*** | 14 | 14 | 22 | 18 | 14 | 14 | 20 | 18 |
| **F** | ***C_min_*** | 14 | 12 | 22 | 18 | 16 | 12 | 24 | 20 |
|  | ***C_max_*** | 18 | 14 | 26 | 24 | 18 | 14 | 28 | 24 |
| **G** | ***C_min_*** | 16 | 18 | 24 | 16 | 16 | 20 | 26 | 16 |
|  | ***C_max_*** | 22 | 24 | 30 | 20 | 24 | 26 | 32 | 20 |
| **H** | ***C_min_*** | 8 | 8 | 16 | 14 | 8 | 8 | 16 | 14 |
|  | ***C_max_*** | 10 | 12 | 24 | 20 | 10 | 10 | 22 | 20 |
| **I** | ***C_min_*** | 16 | 18 | 24 | 16 | 16 | 20 | 26 | 16 |
|  | ***C_max_*** | 22 | 24 | 30 | 20 | 24 | 26 | 32 | 20 |
| **J** | ***C_min_*** | 8 | 8 | 16 | 14 | 8 | 8 | 16 | 14 |
|  | ***C_max_*** | 10 | 12 | 24 | 20 | 10 | 10 | 22 | 20 |

LTA: left tibialis anterior; LLG: left lateral gastrocnemius; LBF: left biceps femoris; LRF: left rectus femoris; RTA: right tibialis anterior; RLG: right lateral gastrocnemius; RBF: right biceps femoris; RRF: right rectus femoris.
